# A supramolecular biomimetic skin combining a wide spectrum of mechanical properties and multiple sensory capabilities

**DOI:** 10.1038/s41467-018-03456-w

**Published:** 2018-03-19

**Authors:** Zhouyue Lei, Peiyi Wu

**Affiliations:** 1 State Key Laboratory of Molecular Engineering of Polymers, Department of Macromolecular Science and Laboratory for Advanced Materials, Fudan University, 200433 Shanghai, China; 2 State Key Laboratory for Modification of Chemical Fibers and Polymer Materials, College of Chemistry, Chemical Engineering and Biotechnology, Center for Advanced Low-Dimension Materials, Donghua University, 201620 Shanghai, China

## Abstract

Biomimetic skin-like materials, capable of adapting shapes to variable environments and sensing external stimuli, are of great significance in a wide range of applications, including artificial intelligence, soft robotics, and smart wearable devices. However, such highly sophisticated intelligence has been mainly found in natural creatures while rarely realized in artificial materials. Herein, we fabricate a type of biomimetic iontronics to imitate natural skins using supramolecular polyelectrolyte hydrogels. The dynamic viscoelastic networks provide the biomimetic skin with a wide spectrum of mechanical properties, including flexible reconfiguration ability, robust elasticity, extremely large stretchability, autonomous self-healability, and recyclability. Meanwhile, polyelectrolytes’ ionic conductivity allows multiple sensory capabilities toward temperature, strain, and stress. This work provides not only insights into dynamic interactions and sensing mechanism of supramolecular iontronics, but may also promote the development of biomimetic skins with sophisticated intelligence similar to natural skins.

## Abstract (web summary)

Biomimetic skin finds wide application in robotics and smart wearable devices but materials mimicking mechanical properties of skin and responding at the same time to multiple stimuli are rarely realized. Here the authors demonstrate a biomimetic hydrogel with multiple sensory capabilities which imitates mechanical properties of natural skin.

## Introduction

Over the past two decades, tremendous evolution of solid-state electronics has occurred and significantly changed our life and society, e.g., the development of super computers and ubiquitous smart devices. However, higher-performance electronic devices have faced limitations of Moore’s law as their dimension reduces to nanoscale^1^. Motivated by this challenge, people have devoted much efforts to developing biomimetic function in the next generation of artificially intelligent devices^2–8^. Nevertheless, there is a fundamental difference in working principles between traditional electronic devices and biological systems. The carriers in electronics are holes and electrons, whose pathways are limited by effective percolation networks. Thus the devices’ electronic conductivity is vulnerable to the geometry deformation. On the contrary, information transduction in biological systems relies on ions’ long-distance transport. Stimuli usually induce ion transport across cell membranes and result in dynamic polarization or depolarization of neurons to transport and process information^1,9^.

Inspired by biological systems, iontronics, as an emerging interdisciplinary technology based on sophisticated control of ions, greatly extends the choices of the next-generation hardware infrastructures for artificial intelligence, i.e, from electronic conductors to bio-inspired ionic conductors^1,10,11^. For instance, hydrogels with microporous structures allow long-distance ion transport and have recently demonstrated their advantages of independent ionic conductivity, biocompatibility, transparency, softness, and stretchablility for biomimetic skin-like iontronics^9,10,12,13^. Accordingly, stretchable chemically crosslinked polyacrylamide hydrogels achieved sensations, including strain^9^, stress^9^, and touch^13^, and were successfully applied as electroluminescent skins for optical signaling^14^, skin-like triboelectric nanogenerator for biomechanical energy harvesting^15^, wearable organic liquid-crystal devices^16^, etc.

However, compared with enormous achievements of electronics witnessed in the past two decades^8^, progress on iontronics is still in its infant stage. Polyacrylamide hydrogel-based devices, on the one hand, provide promising candidates for biomimetic skin-like iontronics, on the other hand, they still lack matched mechanical and sensory performances compared with natural skins. For example, natural skins combine a wide spectrum of mechanical properties and multiple sensory capabilities toward different stimuli, while the traditional chemically crosslinked polyacrylamide hydrogels have limited stretchability, cannot reconfigure themselves to adapt to three-dimensional curved and dynamic surfaces, and fail to autonomously repair themselves after fracture^17^. Moreover, so far very few researches on hydrogel-based skin-like iontronics have realized multiple sensations besides strain and stress^18^. To address the challenges of combining a wide spectrum of mechanical properties and multiple sensory capabilities in artificial skins, supramolecular hydrogels^19–22^ with tunable interactions and dynamic structures may provide a versatile toolbox for the fabrication of skin-like iontronics.

Herein we present a type of supramolecular polyelectrolyte hydrogels to recreate the sophisticated intelligence found in natural skins. The polyelectrolyte networks are facilely synthesized by one-step copolymerization, which achieve unique viscoelasticity and can report external stimuli using ions. By regulating dynamic interactions and energy barriers of the networks, the supramolecular hydrogel realizes a wide spectrum of excellent mechanical properties, including flexible reconfiguration ability, robust elasticity, extremely large stretchability, autonomous self-healability, and recyclability. When the supramolecular hydrogel adopts a sandwiched configuration, it is capable of sensing mechanical deformation based on parallel-plate capacitance and meanwhile distinguishing temperature changes from resistive signals, similar to natural skins which employ different stimuli receptors to sense complex environmental changes.

## Results

### Preparation of the hydrogels

**Fig. 1:**
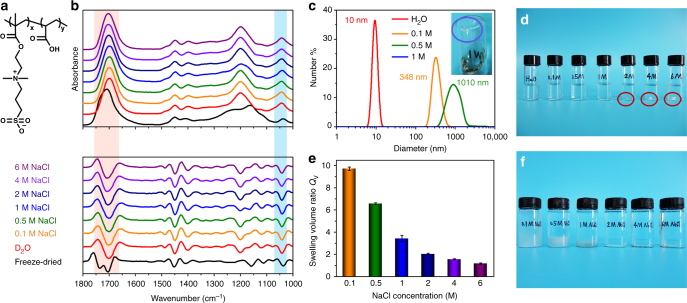
Confirmation of multiple dynamic interactions in the supramolecular networks. **a** The chemical structure of the polyelectrolyte (PAA-co-DMAPS) synthesized in this work. **b** ATR-FTIR spectra (in the regions of 1800–1000 cm^−1^) of the freeze-dried PAA-co-DMAPS copolymer and the supramolecular polyelectrolyte hydrogels equilibrated in D_2_O with different NaCl concentrations and their corresponding second derivative curves. **c** Hydrodynamic diameters of the freeze-dried PAA-co-DMAPS copolymer in aqueous solutions (0.1 wt%) with different NaCl concentrations (0, 0.1, 0.5, and 1 M). The inset photo shows precipitates are observed in 1 M NaCl. **d** A photo of the PAA-co-DMAPS copolymer equilibrated in aqueous solutions (0.1 wt%) with different NaCl concentrations. **e** Swelling volume ratios of the polyelectrolyte hydrogels equilibrated in NaCl aqueous solutions with different concentrations. (error bars: standard deviations). **f** A corresponding photo of the hydrogels in **e**

The supramolecular polyelectrolyte hydrogels were facilely prepared by random copolymerization of acrylic acid (AA) and 3-dimethyl (methacryloyloxyethyl) ammonium propane sulfonate (DMAPS) (Fig. 1a) without chemical crosslinks and further being swollen in NaCl aqueous solutions. Different from tough hydrogels composed of polyampholytes that require a strict charge balance during the copolymerization process^23^, this type of zwitterionic polyelectrolyte hydrogels can be synthesized in a wide range of monomer concentrations, ratios, and ionic strength. And their mechanical properties can be tuned by these factors, as shown in Supplementary Figs. 1–3. To ensure the robustness and avoid bubble generation in the hydrogels, here we chose the sample with the initial monomer concentration of 45% and the monomer mass ratio (AA: DMAPS) of 4:1 for further characterizations.

### Multiple dynamic crosslinks in the supramolecular networks

Firstly, we provide insights into the multiple dynamic crosslinks within the supramolecular polyelectrolyte hydrogels, via infrared spectra, dynamic light scattering (DLS), and swelling measurements. As shown in Fig. 1b, compared with the freeze-dried PAA-co-DMAPS copolymer, C=O stretching bands of PAA and PDMAPS shift to lower frequencies (1701–1709 cm^−1^) in all of the polyelectrolyte hydrogels (D_2_O instead of H_2_O was used as the solvent to eliminate the overlap of H_2_O at around 1640 cm^−1^). They indicate hydrogen bonds between the unionized –COOH as well as ionic associations between –COO^−^ and quaternary ammonium groups^24^, while SO_3_^−^ symmetric stretching bands shift to higher frequencies, from 1038 cm^−1^ in the freeze-dried copolymer to 1042 cm^−1^ in hydrogels, indicating hydration of SO_3_^−^ and increasing ionic associations between SO_3_^−^ and quaternary ammonium groups^25^.

Besides the hydrogen bonds and ionic associations, the addition of NaCl increases hydrophobic interactions, which are demonstrated by the aggregation of PAA-co-DMAPS copolymer (Fig. 1c, d)^26^, smaller swelling volume ratio (Fig. 1e, f), and higher apparent activation energy *E*_a_ (Supplementary Figs. 4–7) of the supramolecular polyelectrolyte networks with enhanced ionic strength. Hydrodynamic diameters of PAA-co-DMAPS linear copolymers (obtained by the dissociation of the supramolecular networks) increase with the addition of NaCl and precipitates are observed in 1, 2, 4, and 6 M NaCl solutions (Fig. 1c, d). Moreover, the increasing hydrophobic interactions result in higher energy barriers of chain motion and thus reinforce the dynamically crosslinked networks with smaller swelling volume ratios (Fig. 1e, f). According to time–temperature superposition curves^27^, *E*_a_ is calculated to be 41 kJ mol^−1^ in polyelectrolyte hydrogels without NaCl and increases to be 59, 66, and 96 kJ mol^−1^ when the hydrogels are equilibrated in 2, 4, and 6 M NaCl solutions, respectively.

### A wide spectrum of mechanical properties

**Fig. 2:**
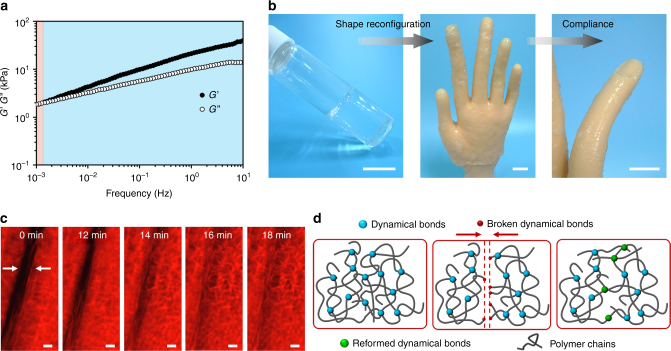
Viscoelasticity, reconfiguration ability, compliance, and autonomous self-healability of the dynamic supramolecular networks. **a** Frequency-dependent storage (*G*’) and loss (*G*”) moduli of the supramolecular polyelectrolyte hydrogel (the monomer mass ratio of AA: DMAPS is 4:1, equilibrated in 4 M NaCl). **b** A photo of the as-prepared transparent hydrogel; it is reshaped to adapt to the irregular surfaces of a prosthetic hand and shows compliance with the prosthetic finger’s locomotion (scale bar: 2 cm). **c** The direct observation of the reconfiguration and autonomous self-healing of the dynamic networks by time-resolved fluorescence microscopic images (scale bar: 20 μm). The white arrows indicate the chains in dynamic networks interdiffuse across the crack. **d** Schematic illustration of the dynamic process

The supramolecular networks with different energy barriers exhibit different time-dependent mechanical properties. Under a 0.5% oscillatory strain, the hydrogel equilibrated in 4 M NaCl displays unique viscoelastic behavior with an elastic–viscous crossover locating at about 10^−3^ Hz at room temperature (Fig. 2a), indicating the hydrogel combines solid-like elasticity and liquid-like plasticity at different time-scales. Therefore, the as-prepared supramolecular hydrogel can be reconfigured to fabricate a thin layer of transparent hydrogel skin for a prosthetic hand (about 90% transmittance in the visible wavelength range, Supplementary Fig. 8). The transparent hydrogel skin shows shape reconfiguration ability to adapt to irregular surfaces and is also compliant with the prosthetic finger’s locomotion (Fig. 2b). This excellent shapeability or reconfiguration ability is similar to some natural creatures, which is important for future soft-bodied robot applications but has never been achieved in previous biomimetic skins. Supplementary Movie 1 records the hydrogel’s wide spectrum of mechanical properties in real time. It not only shows elastic resilience under finger presses and extremely high stretchability but is also able to reconfigure its shapes and heal cracks very fast.

To directly observe the reconfiguration and self-healing behavior of the supramolecular polyelectrolyte networks, we labeled a fractured hydrogel with rhodamine. The fluorescence microscopic images of the hydrogel show an interconnected porous architecture (Fig. 2c), in line with the scanning electron microscopic (SEM) image of a lyophilized sample (Supplementary Fig. 9). We traced the motion of the fractured networks by time-resolved images. As shown in Fig. 2c, when time increases, dynamic hydrogen bonds and ionic associations attract chains to interdiffuse across the crack and engage in new crosslinks. This dynamic process is consistent with the predictive model of autonomously self-healing behavior based on reversible associations^28^. As schematically illustrated in Fig. 2d, along with chain relaxation, the dissociated groups tend to reform bridges and thus repair the networks.

Quantitative characterizations on mechanical properties of this supramolecular polyelectrolyte hydrogel are available in Supplementary Figs. 10–12. It is robust to sustain compression with a compressive modulus of 27.6 kPa (Supplementary Fig. 10), comparable to chemically crosslinked hydrogels and natural skins. Self-healing at the microscopic scale is also evaluated by rheological step-strain measurements. This hydrogel can immediately recover 78.5% of *G*’ and the self-healing behavior can be cycled for several times (Supplementary Fig. 11). As for tensile measurements, both of the pristine and self-healed hydrogels (within 2 h) display a remarkably large elongation of >100 without fracture, indicating the healing efficiency up to almost 100% within 2 h (Supplementary Fig. 12).

We further make a comparison of mechanical properties between this hydrogel and reported fast self-healing hydrogels (within 2 h). As shown in Supplementary Fig. 13, it is worthwhile to note that, with the improvement of stretchability, the elastic modulus usually decreases. For instance, although 2-ureido-4-pyrimidone-based hydrogels can be stretched up to 10,000%, their elastic modulus is smaller than 5 kPa^22^. Hydrogel-based devices with such low modulus may be too weak to resist external variations. Overall, this hydrogel combines robust elasticity, extremely large stretchability, and fast autonomous self-healability, as well as the facile one-step preparation method. This combination has been rarely reported in previous works and is advantageous to develop cost-effective skin-like iontronics adaptable to complex environments.

In addition, this supramolecular polyelectrolyte hydrogel is recyclable. As shown in Supplementary Fig. 14, even if the hydrogel dehydrates, it can recover >90% *G*’ in several dehydration–hydration cycles.

### Multiple sensory capabilities of the hydrogel-based iontronics

**Fig. 3:**
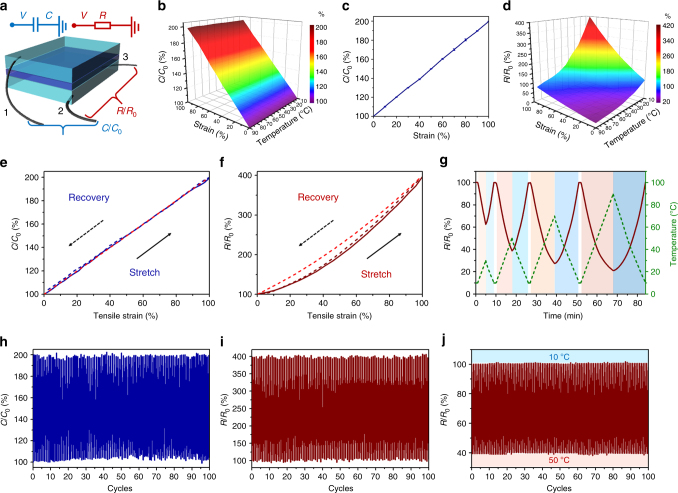
Strain and temperature sensations of the biomimetic skin. **a** Schematic design and two simplified equivalent electrical circuits of the biomimetic skin. **b** The capacitive response of the biomimetic skin upon mutual effect of strain and temperature. **c** The capacitance–strain curve of the biomimetic skin. Data are derived from average values of capacitance at different temperatures in **b**, and error bars (standard deviations) are very small suggesting the capacitive response is insensitive to temperature variation. **d** The resistive response of the biomimetic skin upon mutual effect of strain and temperature. **e** The reversible capacitance–strain curves and the theoretical prediction (red dash line). **f** The reversible resistance–strain curves and the theoretical prediction (red dash line). **g** The reversible resistance–temperature curves. **h** Capacitance–strain cycling curves in the strain range of 0–100%. **i** Resistance–strain cycling curves in the strain range of 0–100%. **j** Resistance–temperature cycling curves in the temperature range of 10–50 °C

Mobile ions in the supramolecular polyelectrolyte hydrogel not only enhance the mechanical performance but also provide the ionic conductivity for information transduction in hydrogel-based iontronics. To recreate the multiple sensory capabilities, here a dielectric elastomer (VHB 4905, 3M) is sandwiched between two ionic–conductive hydrogel layers, which are connected with three metal electrodes. Thus the sandwiched configuration incorporates a parallel-plate capacitive sensor (between electrodes 1 and 2) and an ionic resistive sensor (between electrodes 2 and 3) to mimic different stimuli-receptors in natural skins (Fig. 3a). When the biomimetic skin is stretched at different temperatures, the parallel-plate capacitance is insensitive to temperature variations and displays a nearly linear relationship between the capacitance change and tensile strain (Fig. 3b, c)^9^, whereas resistive signals are thermal-sensitive and can be described as a hyperplane in the “resistance change vs strain vs temperature” space (Fig. 3d). Consequently, the capacitive sensor serves like a mechanoreceptor of natural skins, while the resistive sensor acts like a composite (thermal and mechanical) receptor. When the biomimetic skin is stimulated by both strain and temperature in a complex environment, the capacitive sensor (“mechanoreceptor”) can distinguish the strain stimuli (Fig. 3b, c), and meanwhile the temperature variation can be derived from the resistive hyperplane.

The relationships amoung deformation, temperature, capacitance, and resitance are also predictable. Assuming that the permittivity and volume remain constant as the biomimetic skin deform^9^, when it is stretched by a factor of *λ*, the capacitance *C* follows the linear relationship of *C* = *C*_0_*λ* and the ionic resistance *R* scales as *R* = *R*_0_*λ*^2^ (Fig. 3e, f, *C*_0_, *R*_0_ are the initial capacitance and resistance, respectively, more details are available in Supplementary Information, Supplementary Fig. 15). Moreover, similar to the resistance–temperature relationship found in ionic liquids^29^ and electronic conductors^30^, hydrogel’s ionic resistance decreases with increasing environmental temperature (Fig. 3g). Compared with previously reported skin-like thermal sensors based on metals^31^, self-healing elastomers^30^, electroconductive polymers^32^, nanocomposites^33,34^, etc., this biomimetic skin not only exhibits a relatively high sensitivity but also has a wide temperature sensory range (Supplementary Fig. 16).

In addition to the strain and temperature sensing, this biomimetic skin can also sense the compression stress owing to the geometry changes (Supplementary Fig. 17a). When it is compressed in thickness, its capacitive area correspondingly expands and leads to an increasing capacitance (Supplementary Fig. 17b). Meanwhile, the resistance of the hydrogel layer also increases with the reduction of cross-sectional area and the elongation in length (Supplementary Fig. 17c). Notably, upon applying compression stress, the capacitance *C* and the ionic resistance *R* synchronously increase by a factor of 1/*k*, which is different from the relationship among tensile strain, capacitance, and resistance (more details on the theoretical prediction and experimental data are available in Supplementary Information).

Continuous cycling tests (Fig. 3h–j) and stepwise changes (Supplementary Fig. 18) in the strain and temperature confirm the reliability of this biomimetic skin. Long-term stability is further evaluated. Although this hydrogel can reconfigure its shapes under a dynamic condition, it is quite stable in a static condition. When the hydrogel-based device is placed in a closed environment (neither water evaporation nor epidermis-like elastomer protection, Supplementary Fig. 19), there is a negligible drift (<2%) of the capacitive and resistive signals within 12 h. When the hydrogel exposed to a typical living environment (60% relative humidity, 25 °C), it can keep a relatively stable ionic conductivity for 4 h (Supplementary Fig. 20a). Although long-period dehydration decreases the ionic conductivity, the polyelectrolyte xerogel still has an ionic conductivity of around 2 × 10^−5^ S cm^−1^ after 20 days. Therefore, the dehydrated device maintains the thermo-sensitive resistance (Supplementary Fig. 20b) and stable capacitive signals (Supplementary Fig. 20c). Inspired by the structures of mammalian skins, people can introduce some epidermis-like elastomers to protect this supramolecular skin^9,35^ (Supplementary Fig. 21) or recycle it from the reversible dehydration–hydration process (Supplementary Figs. 14 and 22).

### Applications as a type of multifunctional biomimetic skin

**Fig. 4:**
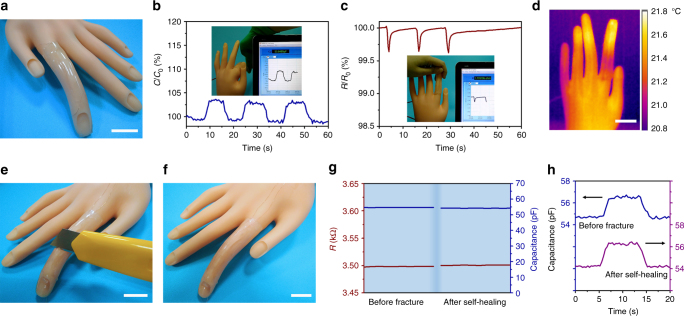
Applications in strain and temperature sensing and self-healability of the biomimetic skin. **a** A photo of the biomimetic skin attached to a prosthetic finger with the assistance of VHB tapes. **b** Capacitive signals monitor the finger’s movements. The inset photo is derived from Supplementary Movie 2. **c** Resistive signals when a person’s hand contacts the prosthetic finger. The inset photo is derived from Supplementary Movie 3. **d** An infrared image of the prosthetic hand after the removal of the person’s hand. **e** A photo of a biomimetic skin whose hydrogel layer is cut into half. **f** A photo of the biomimetic skin after self-healing. **g** The resistance and capacitance of the biomimetic skin before fracture and after self-healing. **h** The capacitance changes of the biomimetic skin before fracture and after self-healing when it is applied to detect a prosthetic finger’s bending–straightening movement (the capacitance increases when the finger bends and decreases when it straightens). (Scale bar: 2 cm)

When the biomimetic skin is attached to a prosthetic finger with the assistance of an epidermis-like VHB tape (Fig. 4a), it enables the prosthetic finger to sense strain and temperature stimuli through different stimuli-receptors, i.e., capacitive and resistive sensors, respectively. For instance, it can record the finger’s bending–straightening movement information based on capacitance changes during deformation (Fig. 4b, Supplementary Movie 2). When a person’s hand touches the prosthetic finger, the biomimetic skin can also sense the temperature increase with the real-time resistance decrease (Fig. 4c, Supplementary Movie 3). According to the resistance–temperature relationship around room temperature, temperature increases about 0.3 °C, roughly in line with the average temperature variation determined by an infrared image of the prosthetic hand, which is taken immediately after the removal of the human hand (Fig. 4d).

Besides strain and temperature sensations, this biomimetic skin has similar self-healability compared to natural skins. When the ionic-conductive hydrogel layer of the biomimetic skin is cut into half (Fig. 4e), it can autonomously repair the crack after being brought into contact (Fig. 4f). Within 20 min, the ionic resistance and capacitance can be restored with only about 0.04 and 0.9% drift, respectively (Fig. 4g). The self-healing biomimetic skin is able to detect the finger’s movement and shows similar capacitance changes before fracture and after self-healing (Fig. 4h). In addition, the capacitance–strain, resistance–strain, and resistance–temperature relationships recover as the self-healing of the ionic-conductive hydrogel (Supplementary Fig. 23). However, if the commercial dielectric layer is also damaged, the recovery of the biomimetic skin will be limited by the healing efficiency of the dielectric layer. Since here the VHB tapes can glue itself after being cut into half, thus the ionic resistance and capacitance can also recover within 20 min (Supplementary Fig. 24).

Comprehensive performance of this biomimetic skin is compared with that of natural skins and other artificial skins in Supplementary Fig. 25. This platform combines a wide spectrum of mechanical properties and multiple sensations, which has rarely been achieved in previous works, e.g., typical artificial skins based on stretchable silicon^5^, polyacrylamide hydrogels^9^, and self-healing elastomers^36^.

## Discussion

Although the development of iontronics is still in its infant stage, here we use supramolecular hydrogels to fabricate biomimetic skin-like iontronics and demonstrate their advantages of mechanical adaptability to dynamic environments, multiple sensations toward external stimuli, and effective interactions with human beings. By providing insights into dynamic nature of the supramolecular polyelectrolyte hydrogels, we show a facile method to imitate the mechanical properties of natural skins, which combines robust elasticity, extremely large stretchability, autonomous self-healability, recyclability, and even flexible reconfiguration ability. Moreover, ion transport within the polyelectrolyte networks enable the skin-like iontronics to sense external stimuli, including strain, temperature, and stress. To the best of our knowledge, it is the first example that achieves sophisticated intelligence in biomimetic skin-like materials combining such a wide spectrum of mechanical properties and multiple sensations.

Since the supramolecular biomimetic skin has remarkable reconfiguration ability and their ion transport has no limitation of percolation pathways, we believe this work provides an important alternative to construct deformable sensory systems in the next generation of soft intelligent robots and smart wearable devices for Internet of Things applications. The facileness of the proposed method makes the biomimetic skin suitable for large-scale fabrication and potential incorporation into advanced processing techniques, such as additive manufacturing. It may also inspire people to harness distinctive but complementary advantages of supramolecular hydrogels to address some challenges faced by electronics.

## Methods

### Materials

DMAPS and AA were purchased from Sigma-Aldrich Co. Ammonium persulfate (APS), NaCl, and rhodamine were purchased from Aladdin Chemical Co. AA monomer was purified by vacuum distillation. APS was recrystallized before use. Other reagents were used without further purification.

### Preparation of supramolecular hydrogels

Supramolecular polyelectrolyte hydrogels were fabricated by the one-step random copolymerization of AA and DMAPS monomers. The monomer ratios and concentrations were tunable. The polymerization was initiated by APS (0.2 mol%, relative to the total monomer concentration) and proceeded at 50 °C for 24 h. After polymerization, they were first washed with deionized water (~ 10 °C) to remove any residual chemicals and then immersed in NaCl aqueous solutions with different concentrations at 80 °C for 2 days and further equilibrated at room temperature for 5 days.

### Characterization of the multiple dynamic crosslinks

Infrared spectra were recorded on a Nicolet 6700 spectrometer using attenuated total reflectance (ATR) method with a diamond ATR crystal as the window material. DLS measurements were carried out on a Zetasizer Nano system (Malvern) with a scattering angle of 173°. The copolymer concentration is 0.1 wt%. For swelling measurements, the fresh prepared polyelectrolyte hydrogels were immersed in NaCl aqueous solutions with different concentrations at 80 °C for 2 days and further equilibrated at room temperature for 5 days. Swelling volume ratio *Q*_v_ is defined as the ratio of the volume at swelling equilibrium *V* to that in the as-prepared state *V*_0_, *Q*_v_ = *V*/*V*_0_.

### Characterization of the mechanical properties

The rheological behavior was investigated by a HAAKE MARS modular advanced rheometer using 25 mm parallel plate. Dynamic frequency sweep measurements were conducted at 25 °C in an oscillation mode with a fixed oscillatory strain of 0.5%. Time–temperature superposition curves were obtained by the dynamic frequency sweep at different temperatures with a fixed oscillatory strain of 0.5%. We used 35 °C as the reference temperature. Strain sweep measurements of the supramolecular polyelectrolyte hydrogel were performed from 0.1 to 1000% and back to 0.1% strain at the frequency of 1 Hz. Continuous step-strain measurements were conducted at high-amplitude oscillatory (*γ* = 1000%) and low-amplitude oscillatory (*γ* = 0.1%) at the frequency of 1 Hz (25 °C). Compression and tensile tests were performed on a universal mechanical test machine (CMT4104) at 25 °C.

### Microscopic observation of the supramolecular networks

For direct observation of the dynamic behavior of the supramolecular polyelectrolyte networks via fluorescence microscopic images (Leica DM2500P, Germany), the hydrogel was labeled with rhodamine through electrostatic interactions. It was first immersed in 4 M NaCl aqueous solution containing 0.01 g L^−1^ rhodamine. After 12 h, the hydrogel was washed with a large amount of deionized water (~ 10 °C) to remove excess rhodamine. We cut the hydrogel into two pieces and then put them together to observe the self-healing process of the fractured networks. SEM image of a freeze-dried sample was taken on a Zeiss Ultra 55 microscope.

### The transmittance measurement

The transmission spectrum of the hydrogel with a thickness of 1 cm was recorded on a Lambda 750 spectrophotometer (Perkin-Elmer).

### Recyclability assessments

The hydrogel was dried in air and swelled in 4 M NaCl for several dehydration–hydration cycles, and the recyclability was quantitatively assessed by the recovery of *G*’ (at the frequency of 1 Hz and a fixed oscillatory strain of 0.5%) and the resistance (*R*/*R*_0_, %).

### Characterization of the hydrogel-based biomimetic skin

The biomimetic skin was based on the hydrogel, which is equilibrated in 4 M NaCl with the initial monomer concentration of 45% and the monomer mass ratio (AA: DMAPS) of 4:1. Infrared images of the biomimetic skin were recorded by an infrared thermal camera (FLIR T630SC). The capacitive and resistive signals were recorded on an LCR meter (TH2830) controlled by a LabView program. The LCR meter was used to simultaneously detect capacitance/resistance changes when the hydrogel-based iontronics were stimulated by strain, temperature, and stress. Heating and cooling cyclic tests are performed with a Peltier temperature controller. Unless otherwise stated, we introduced an epidermis-like elastomer (VHB 4905, 3M) to protect the supramolecular skin when measuring capacitance and resistance upon strain, temperature, and stress stimuli^9^. Besides, long-term stability of the hydrogel-based iontronics was measured in a BPHJ-120AF temperature and humidity test chamber at the certain condition (Shanghai Bluepard Instruments Co., Ltd).

### Data availability

The data that support the findings of this study are available from the corresponding author upon reasonable request.

## Electronic supplementary material

Supplementary(PDF 1483 kb)

Description of Additional Supplementary Files(PDF 52 kb)

Supplementary Movie 1

Supplementary Movie 2

Supplementary Movie 3
