# Supplementary material for: A supramolecular biomimetic skin combining a wide spectrum of mechanical properties and multiple sensory capabilities
Source: Nat Commun. 2018 Mar 19;9:1134. doi: 10.1038/s41467-018-03456-w (PMC5859265; doi:10.1038/s41467-018-03456-w)
Supplement: Supplementary file 1 — Supplementary(PDF 1483 kb) [file 41467_2018_3456_MOESM1_ESM.pdf]

A supramolecular biomimetic skin combining a wide spectrum of mechanical properties and multiple sensory capabilities

Zhouyue Lei[†], and Peiyi Wu*[†‡]

[†]State Key Laboratory of Molecular Engineering of Polymers, Department of Macromolecular Science and Laboratory for Advanced Materials, Fudan University, Shanghai 200433, China

[‡]State Key Laboratory for Modification of Chemical Fibers and Polymer Materials, College of Chemistry, Chemical Engineering and Biotechnology, Center for Advanced Low-Dimension Materials, Donghua University, Shanghai 201620, China

E-mail: peiyiwu@fudan.edu.cn

Supplementary Note 1. Effect of monomer ratio

Supplementary Fig. 1 shows the effect of monomer ratios on the rheological behavior. Elastic supramolecular networks can be formed in a wide range of monomer ratio. When the monomer mass ratio of AA: DMAPS is increased from 1:1 to 5:1, the storage modulus (G') slightly increases. However, further increasing the monomer mass ratio results in bubbles within the supramolecular polyelectrolyte hydrogel. Therefore, we chose the sample with the monomer mass ratio of 4:1 for further characterizations.

Supplementary Note 2. Effect of monomer concentrations

Supplementary Fig. 2 shows the effect of initial monomer concentrations on the rheological behavior. Elastic supramolecular networks can be formed in a wide range of monomer concentrations. When the monomer concentration decreases to 25%, the loss modulus (G'') is close to the storage modulus (G'). The supramolecular polyelectrolyte hydrogel displays viscous liquid-like feature at room temperature. When the initial monomer concentration is increased, G' significantly increases and the networks exhibit predominantly elastic feature. Whereas the concentration is above 50%, it tends to generate bubbles within the hydrogel. People can tailor the mechanical properties according to their demands. Here we chose the sample with the initial monomer concentration of 45% and the monomer mass ratio (AA: DMAPS) of 4:1 for further characterizations.

Supplementary Note 3. Effect of ionic strength

Supplementary Fig. 3 shows the effect of NaCl concentrations on the rheological behavior. After being equilibrated in NaCl aqueous solutions with different concentrations, the supramolecular polyelectrolyte hydrogels display different rheological properties. The as-prepared polyelectrolyte hydrogels dissolve in water, or form viscous polymer liquids in NaCl aqueous solutions with relatively low ionic concentrations (0.1-1 M). Higher salt concentrations (2-

6 M) result in higher elasticity of the hydrogels, indicating the chain motion is restricted at high ionic strength. Therefore, the viscoelasticity of the supramolecular polyelectrolyte hydrogels can be simply regulated by the salt concentrations (ionic strength).

When the monomer mass ratio (AA:DMAPS) in the range of 1:1 to 5:1, the monomer concentration locates at 25-50%, and the NaCl concentration is adjusted to be 2-6 M, the supramolecular polyelectrolyte hydrogels are dominantly elastic and their moduli are comparable to those of permanently-crosslinked hydrogels. Increasing monomer concentration, monomer ratio of AA: DMAPS and/or ionic strength, results in higher elastic moduli (G'). However, there is no covalent crosslink within the networks as they completely dissolve in water at high temperature (80 ^{o}C) after 2 days.

Supplementary Note 4. Apparent activation energy E_a of the supramoecular networks with different ionic strength

Supplementary Fig. 4 shows the dynamic behavior of the supramolecular polyelectrolyte hydrogel without the addition of NaCl at different temperatures and frequencies follows the principle of time-temperature superposition well. The supramolecular polyelectrolyte hydrogel exhibits solid-like behavior (G'>G'') in the frequency range of 0.1-100 Hz and the temperature range of 5-50 ^{o}C. At higher temperature and/or lower frequency (longer time), the value of G'' is close to that of G', which indicates the equilibrium of viscosity and elasticity can be shifted by temperature, time or apparent activation energy of the polyelectrolyte networks.

The apparent activation energy E_a is obtained from the Arrhenius equation, $a_T = \mathrm{A}e^{\mathrm{E_a}/\mathrm{R}T}$, in which a_T is the horizontal shift factor, R is the ideal gas constant, and A is a constant.[1] The apparent activation energy value (41 kJ mol^{-1}) is calculated from the slope of the curve in Supplementary Fig. 4b. The vertical shift factor (b_T) which is very close to 1, confirming the thermorheologically simple behavior of the supramolecular polyelectrolyte

hydrogel (Supplementary Fig. 4c).[2]

Supplementary Fig. 4-7 show that the increasing ionic strength results in higher apparent activation energy of the supramolecular polyelectrolyte hydrogels. This phenomenon is consistent with the rheological behavior shown in Supplementary Fig. 3 and smaller swelling volume ratios shown in Figure 1e and f. Higher ionic strength enhances the hydrophobic interactions, as confirmed in Figure 1c and d, thus constrains the polymer chains' motion and reinforces the polyelectrolyte networks with higher apparent activation energy. We chose the hydrogel equilibrated in 4 M NaCl aqueous solution for further characterizations and preparing multifunctional biomimetic skin. People can tailor the mechanical properties of the supramolecular polyelectrolyte hydrogels based on their demands.

Supplementary Note 5. Transmittance of the supramolecular polyelectrolyte hydrogel

Supplementary Fig. 8 shows the hydrogel reaches about 90% transmittance in the visible wavelength range. This is advantageous to develop transparent wearable iontronics that are invisible to naked eyes.

Supplementary Note 6. A Scanning electron microscopy (SEM) image of the lyophilized networks

Supplementary Fig. 9 shows an interconnected porous architecture of the supramolecular polyelectrolyte hydrogel networks, which is similar to the architecture labeled with rhodamine shown in fluorescence microscopic image (Figure 2c). According to previous reports, lyophilization might cause the shrinkage of the networks, however, the lyophilized sample retains the integration and porosity. It indicates the relatively high strength of the supramolecular networks.

Supplementary Note 7. Compression stress–strain curves of the supramolecular polyelectrolyte hydrogel

Supplementary Fig. 10 shows the supramolecular polyelectrolyte hydrogel is robust to sustain compressions. The compression stress-strain curves were recorded at a deformation rate of 1 $mm \cdot min^{-1}$. The compressive modulus is calculated to be 27.6 kPa by linear fitting (red dash line) of the slope in Supplementary Fig. 10b.

Supplementary Note 8. Strain sweep and continuous step-strain measurements of the supramolecular hydrogel

Supplementary Fig. 11 shows the self-healing behavior of the hydrogel, which can recover 78.5% of G' immediately and the self-healing behavior could be cycled for several times.

Supplementary Note 9. True tensile stress–strain curves of the supramolecular hydrogels

Supplementary Fig. 12 shows the supramolecular polyelectrolyte hydrogel is extremely stretchable and has excellent self-healability. The tensile stress-strain curves were recorded at a deformation rate of 100 $mm \cdot min^{-1}$. The curve of the self-healing sample was recorded within 2 hours. Here we use the true stress instead of the nominal stress since the hydrogels have very large deformation. On the basis of the assumption that the hydrogels are incompressible, the true stress is calculated from the nominal stress using the expression $\sigma = S \cdot \lambda$.[3]

Supplementary Note 10. A comparison of mechanical properties

Our hydrogel is capable of being stretched more than 10000% of its original length without fracture, which is at the highest level of stretchability reported in literatures. Commercial elastomers usually have an elongation at break less than 1000% of their original length. As for highly stretchable hydrogels reported

previously in the literature, only very few examples display elongations more than 10000% of their original lengths, for example, a UPyHCBA-based hydrogel[3] and a Pluronic F127 hydrogel[4]. Here we make a comparison between this hydrogel and previously-reported stretchable and fast self-healing (within 2 hours) hydrogels. As shown in Supplementary Fig. 13, most of the hydrogels reported for high mechanical performance, i.e., ACC/PAA/alginate hydrogels,[5] PDMA/SDS hydrogels,[6] and Agarose/PVA double-network hydrogels (0.5 wt% agarose)[7], cannot reach such high stretchability. With the improvement of stretchability and self-healability, the elastic modulus of previously reported hydrogels usually decreases. It is worthwhile to note that, although the UPyHCBA-based hydrogel can be stretched up to 10000%, the elastic modulus of UPyHCBA-based hydrogel is smaller than 5 kPa, and the self-healing time of the Pluronic F127 hydrogel requires at least 24 hours for 85% self-healing efficiency. Overall, our hydrogel displays a wide spectrum of excellent mechanical properties including robust elasticity, extremely large stretchability, and fast autonomous self-healability.

Supplementary Note 11. Recovery of the shape and modulus of the supramolecular hydrogel

Supplementary Fig. 14 shows the excellent recyclability of the supramolecular polyelectrolyte hydrogel (> 90% recovery of G' in ten dehydration-hydration cycles). The excellent recyclability of the hydrogel is advantageous to practical applications and extend its life-time.

Supplementary Note 12. Theoretical prediction of capacitance/resistance-strain signals

As shown in Supplementary Fig. 15, the theoretical prediction of the capacitance is derived from a parallel-plate configuration, $C = \varepsilon S/4\pi k d$ (C is the capacitance; ε is the dielectric constant of the dielectric layer; k is the electrostatic constant; S is the effective area of the conductive layer; d is the

thickness of the dielectric layer). Assuming that the device volume and permittivity remain constant,[8] when the device is stretched by a factor, $\lambda=L/L_0$, the area ($S = W L$) of the ionic-conductive hydrogel layer scales by the factor of $\sqrt{\lambda}$, and the thickness of the dielectric layer (d) scales by the factor of $\frac{1}{\sqrt{\lambda}}$. Consequently, the capacitance C is supposed to scale as $C = C_0 \lambda$ (C_0 is the initial capacitance).

Besides, the ionic resistance of the hydrogel layer is given by $R = \rho L/A$, in which ρ is the resistivity, L and A are the geometry factors corresponding to the length and cross-sectional area respectively. When this device is stretched by a factor, $\lambda=L/L_0$, the cross-sectional area ($A = W T$) of the ionic-conductive hydrogel layer scales by the factor of $\frac{1}{\lambda}$, and the length scales by the factor of λ. Therefore, the resistance R scales as $R = R_0 \lambda^2$ (R_0 is the initial resistance).

Supplementary Note 13. A comparison of thermal sensory capability

We compare thermal sensory capability of our supramolecular biomimetic skin with other works published previously in literatures. As shown in Supplementary Fig. 16, the sensitivity of this supramolecular skin is higher than that of gold sensors[9], printable electroconductive temperature sensors (CNT-PEDOT:PSS)[10], self-healing conductive elastomers based on SWCNTs[11], polypyrrole sensors[12], etc, and comparable to some of electroconductive nanocomposites[13,14]. Although percolation-type thermistors offer a very high ΔR/R value which results in much higher sensitivity, such resistance changes typically occur in a very narrow temperature range (within 10 °C).[14,15] Whereas, our platform has a broad temperature sensory range up to 80 °C, wider than that of the gold sensors[9], and some of the electroconductive nanocomposites[12,13].

Supplementary Note 14. Theoretical prediction and experimental data of capacitance/resistance-stress signals

As shown in Supplementary Fig. 17a, when the device is compressed by a factor of k, the area ($S = W L$) of the ionic-conductive hydrogel layer scales by the factor of $\frac{1}{k}$, while the thickness change of the the dielectric layer is negligible since it is much thinner and has much higher modulus than the hydrogel layers. As a result, the capacitance C is supposed to scale as $C = C_0 \frac{1}{k}$.

Meanwhile, the cross-sectional area ($A = W T$) of the ionic-conductive hydrogel layer scales by the factor of $\sqrt{k}$ and the length scales by the factor of $\frac{1}{\sqrt{k}}$. Consequently, the resistance R scales as $R = R_0 \frac{1}{k}$.

On the basis of the theoretical prediction, the capacitance C and the ionic resistance R synchronously increase by a factor $\frac{1}{k}$ when compression stress is applied on the hydrogel-based iontronics.

Supplementary Fig. 17b and c record the capacitance-stress and resistance-stress relationships when the biomimetic skin is compressed. Their trends are in line with the theoretical prediction.

Supplementary Note 15. Stepwise changes in the strain and temperature

As shown in Supplementary Fig. 18, this supramolecular biomimetic skin is capable of maintaining a changed state and recovering the initial state after the removal of mechanical stimuli.

Supplementary Note 16. Stability of the hydrogel-based iontronics in a closed environment

As shown in Supplementary Fig. 19, there is a negligible drift (less than 2%) of the capacitance and resistance signals in a closed environment within 12 hours (neither water evaporation nor epidermis-like VHB protection), suggesting the hydrogel-based iontronics' geometric stability. It is worthwhile to note that,

although the supramolecular polyelectrolyte hydrogel has reconfiguration capability in a dynamic condition, and can self-heal the cracks when fractured hydrogels or different parts of a hydrogel are brought to intimately contact with each other, the individual hydrogel-based device is quite stable in a static condition. Moreover, this supramolecular polyelectrolyte hydrogel has a compressive modulus of 27.6 kPa (Supplementary Fig. 10), which is comparable to chemically crosslinked hydrogels and natural skins. In the Supporting Movie S1, it also shows elastic resilience under finger presses. We believe the combination of the elastic resilience (stability) and reconfiguration capability and is owing to the multiple dynamic interactions within the hydrogel. On the one hand, the multiple interactions improve the elasticity (stability) of the hydrogel, and on the other hand, their dynamic nature allows the reversible associations of the hydrogel networks. This combination has been rarely reported and is one of the advantages of the supramolecular biomimetic skin.

Supplementary Note 17. Stability of the hydrogel-based iontronics in a typical living environment

As shown in Supplementary Fig. 20a, without protection, the supramolecular hydrogel can maintain a relatively stable ionic resistance in a typical living environment for 4 hours (60% relative humidity at room temperature, only about 10% drift of resistance). In a long period, dehydration decreases the ionic conductivity of the hydrogel. Fortunately, even the hydrogel completely changes into a xerogel, the polyelectrolyte xerogel still has an ionic conductivity of around 2×10^{-5} S cm^{-1}. Therefore, it maintains thermo-sensitive resistance (Supplementary Fig. 20b, the sensitivity of temperature sensing is even improved) and stable capacitive signals (Supplementary Fig. 20c).

Supplementary Note 18. Stability of the hydrogel-based iontronics with epidermis-like VHB protection

Inspired by the structures of mammalian skins, in which the elastomer-like

epidermis can effectively prevent the hydrogel-like body from dehydration,[16] here we use VHB tapes (3M, 4905) to mimic the epidermis. With the protection of epidermis-like VHB tapes, both of the capacitive and resistive sensing are stable for a trial period of 20 days (Supplementary Fig. 21). Overall, although long-period dehydration has some influence on the supramolecular hydrogel, people can introduce some epidermis-like elastomers to prevent dehydration.

Supplementary Note 19. Recovery of the resistance of the supramolecular polyelectrolyte hydrogel

Supplementary Fig. 22 shows the excellent recyclability of the supramolecular polyelectrolyte hydrogel with nearly 100% recovery of the resistance in ten dehydration-hydration cycles. As the one-step copolymerization method is very facile, the supramolecular biomimetic skin is cost-effective, and the dehydration-hydration process is reversible, we believe this work is advantageous to the development of hardware infrastructures for future artificial intelligence and Internet of Things (IoT) applications.

Supplementary Note 20. The restoration of sensory capabilities of a fractured biomimetic skin

When the ionic-conductive hydrogel layer of the biomimetic skin is cut into half and the dielectric layer is not damaged, the hydrogel can autonomously repair the crack after being brought into contact (Fig. 4e, f). The ionic resistance and capacitance can be restored within 20 min. And the capacitance-strain, resistance-strain and resistance-temperature relationships also recover, similar to the relationships before fracture.

If the commercial dielectric layer is also damaged, the restoration of sensory capabilities of the biomimetic skin will be limited by the healing efficiency of the dielectric layer. Since here the VHB tapes can glue itself after being cut into half, thus the ionic resistance and capacitance can also recover within 20 min, with only about 0.2 % and 3% difference, respectively. However, the perfect

restoration of the sensory capabilities requires autonomously self-healing dielectric materials to replace the current commercial one.

Supplementary Note 21. A comparison of mechanical properties and sensations

We compare the comprehensive performance of our platform with natural skins and other artificial skins. As shown in Supplementary Fig. 25, this platform combines a wide spectrum of mechanical properties and multiple sensations, which has been rarely achieved in previous works. For instance, typical artificial skins based on stretchable silicon[17], polyacrylamide hydrogels[8] and self-healing elastomers[18] have their unique advantages but fail to cover such wide properties.

Supplementary Figure 1

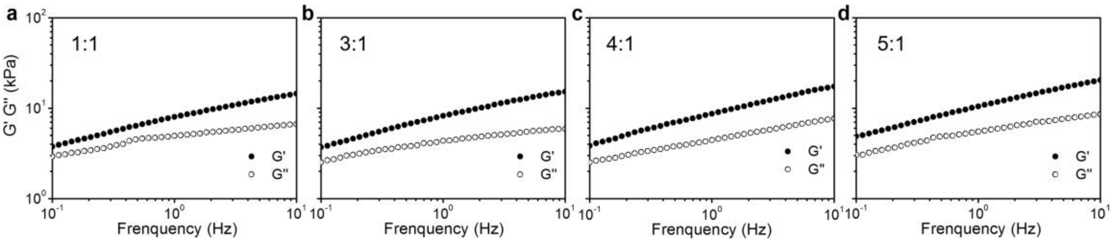

Supplementary Figure 1. Monomer ratio effect on the rheological behavior. The monomer mass ratios of AA: DMAPS are 1:1, 3:1, 4:1 and 5:1 in (a), (b), (c) and (d), respectively. The monomer concentration in precursor aqueous solutions is fixed at 45 wt% without the addition of NaCl.

Supplementary Figure 2

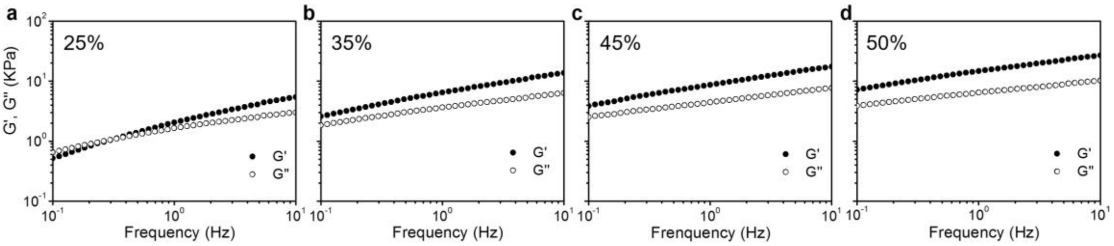

Supplementary Figure 2. Monomer concentration effect on the rheological behavior. The monomer mass concentrations of the precursor aqueous solutions are 25%, 35%, 45% and 50% in (a), (b), (c) and (d), respectively. The monomer mass ratio of AA: DMAPS is fixed at 4:1 without the addition of NaCl.

Supplementary Figure 3

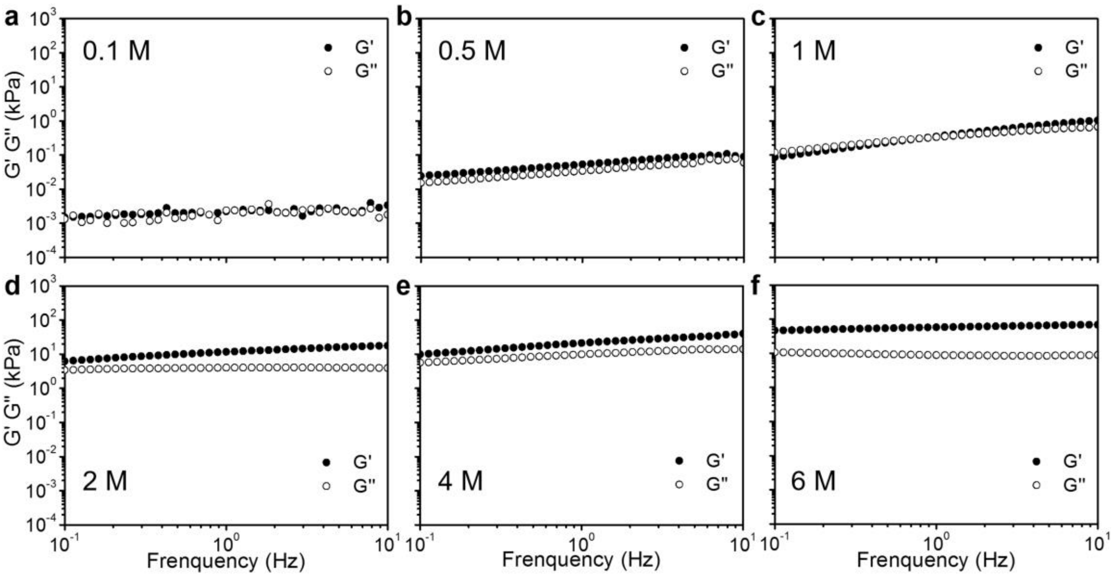

Supplementary Figure 3. NaCl concentration effect on the rheological behavior. The as-prepared polyelectrolyte hydrogels (the monomer concentration is 45%, and the monomer mass ratio of AA: DMAPS is 4:1) are further immersed in NaCl aqueous solutions with different concentrations at 80 °C for 2 days and equilibrated at room temperature for 5 days. The NaCl concentrations are 0.1, 0.5, 1, 2, 4 and 6 M in (a), (b), (c), (d), (e), and (f), respectively.

Supplementary Figure 4

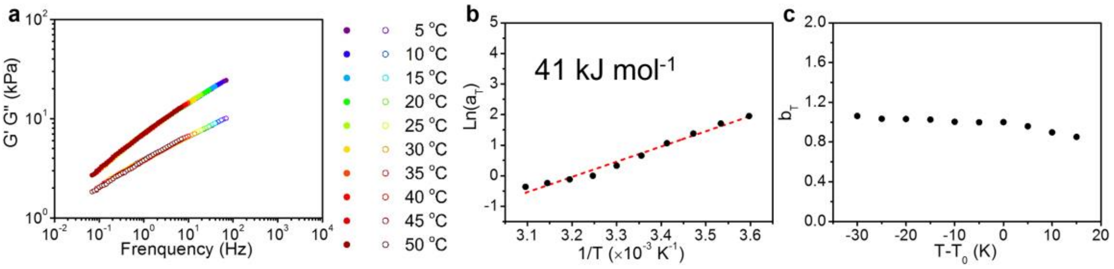

Supplementary Figure 4. Dynamic mechanical behavior of the as-prepared supramolecular networks without the addition of NaCl. (a) Classical time-temperature superposition shifts at a reference temperature of 35 °C. (b) Time−temperature horizontal shift factor (a_T) derived from Arrhenius equation. (c) Vertical shift factor (b_T) as a function of temperature.

Supplementary Figure 5

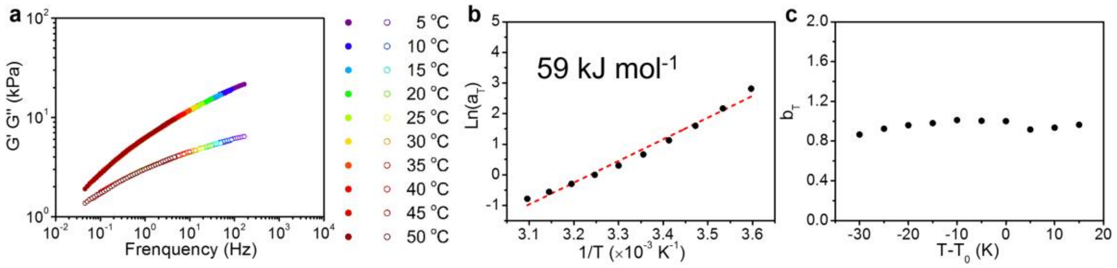

Supplementary Figure 5. Dynamic mechanical behavior of the supramolecular networks equilibrated in 2 M NaCl aqueous solution. (a) Classical time-temperature superposition shifts at a reference temperature of 35 °C. The apparent activation energy value (59 kJ mol^{-1}) is calculated from the slope of the curve. (b) Time−temperature horizontal shift factor (a_T) derived from Arrhenius equation. (c) Vertical shift factor (b_T) as a function of temperature.

Supplementary Figure 6

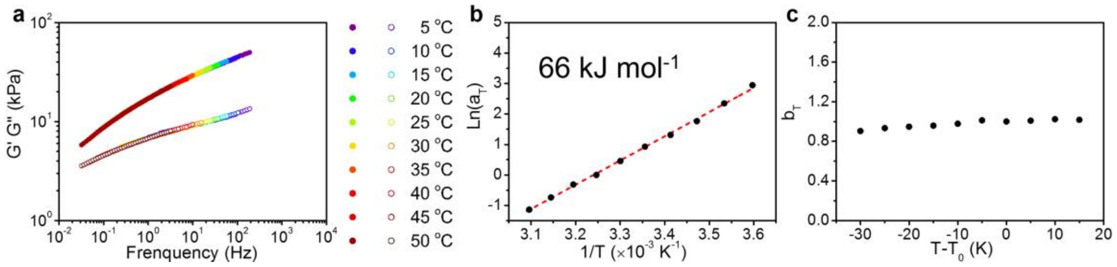

Supplementary Figure 6. Dynamic mechanical behavior of the supramolecular networks equilibrated in 4 M NaCl aqueous solution. (a) Classical time-temperature superposition shifts at a reference temperature of 35 °C. The apparent activation energy value (66 kJ mol^{-1}) is calculated from the slope of the curve. (b) Time−temperature horizontal shift factor (a_T) derived from Arrhenius equation. (c) Vertical shift factor (b_T) as a function of temperature.

Supplementary Figure 7

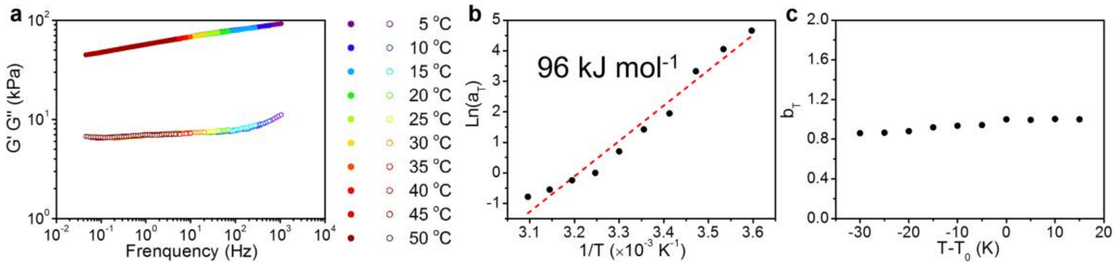

Supplementary Figure 7. Dynamic mechanical behavior of the supramolecular networks equilibrated in 6 M NaCl aqueous solution. (a) Classical time-temperature superposition shifts at a reference temperature of 35 °C. The apparent activation energy value (96 kJ mol^{-1}) is calculated from the slope of the curve. (b) Time−temperature horizontal shift factor (a_T) derived from Arrhenius equation. (c) Vertical shift factor (b_T) as a function of temperature.

Supplementary Figure 8

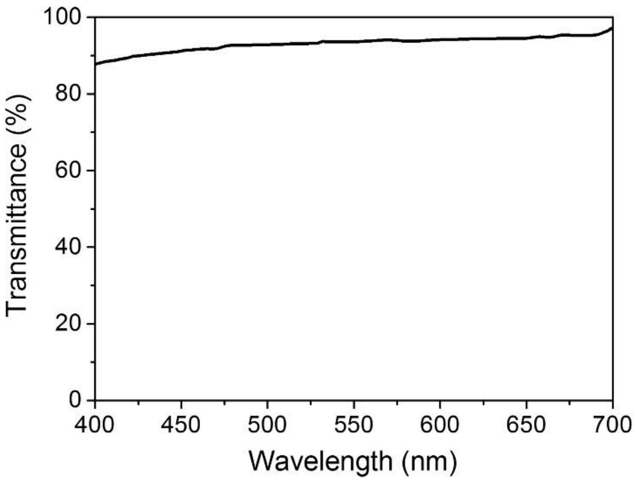

Supplementary Figure 8. The transmittance of the hydrogel in the visible wavelength range of 400-700 nm.

Supplementary Figure 9

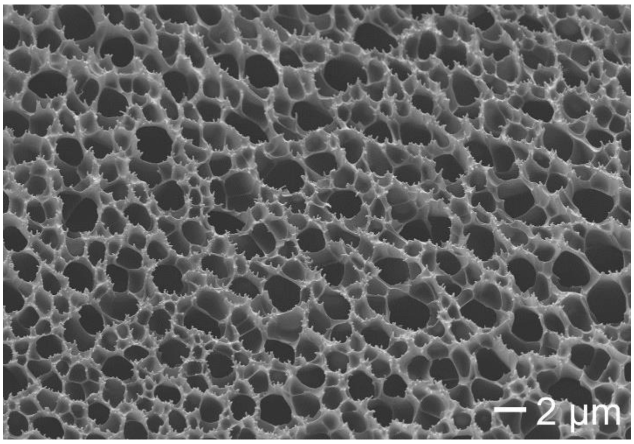

Supplementary Figure 9. An SEM image of the lyophilized networks.

Supplementary Figure 10

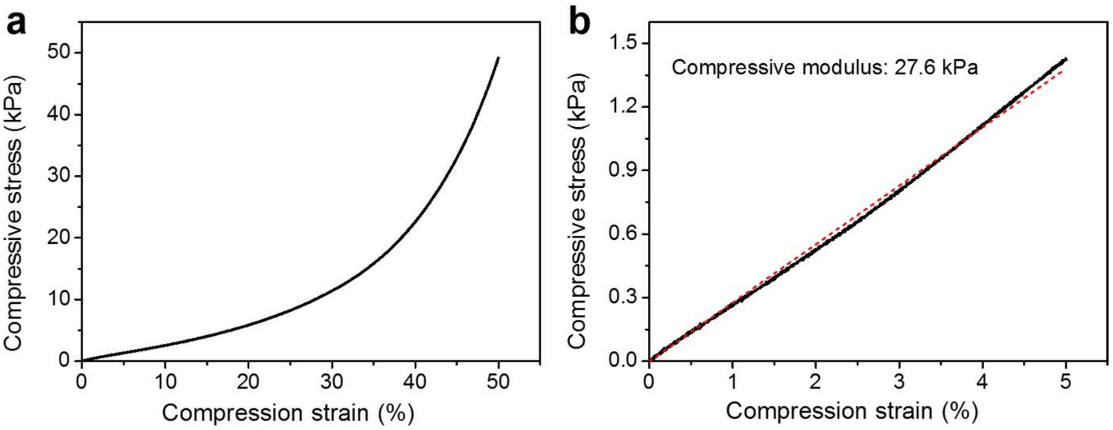

Supplementary Figure 10. The compression stress-strain curves in the strain ranges of (a) 0-50% and (b) 0-5%.

Supplementary Figure 11

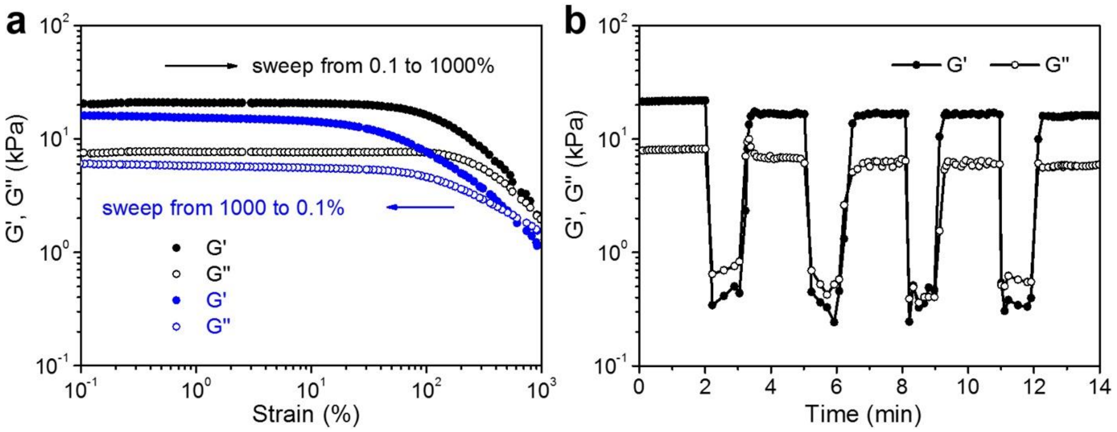

Supplementary Figure 11. Rheological analysis of the self-healing behaviors. (a) Strain sweep measurements of the hydrogel from 0.1% (G' = 20.5 kPa) to 1000% and back to 0.1% (G' = 16.1 kPa) at the frequency of 1 Hz. (b) Continuous step-strain measurements of the supramolecular polyelectrolyte hydrogel at high-amplitude oscillatory (γ = 1000%) and low-amplitude oscillatory (γ = 0.1%) at the frequency of 1 Hz (25 °C).

Supplementary Figure 12

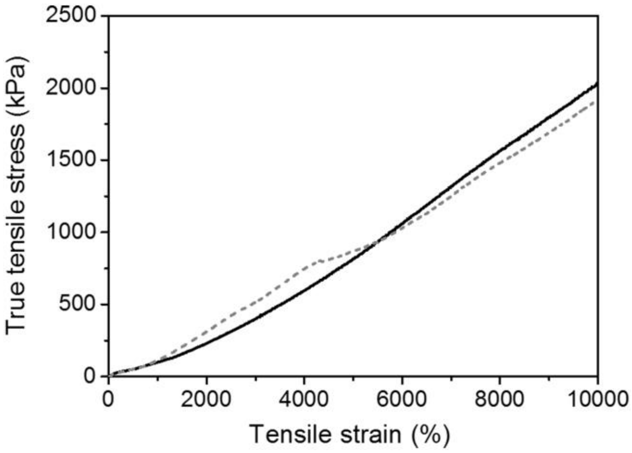

Supplementary Figure 12. The true tensile stress–strain curves of the pristine (solid line) and self-healed hydrogels (dash line).

Supplementary Figure 13

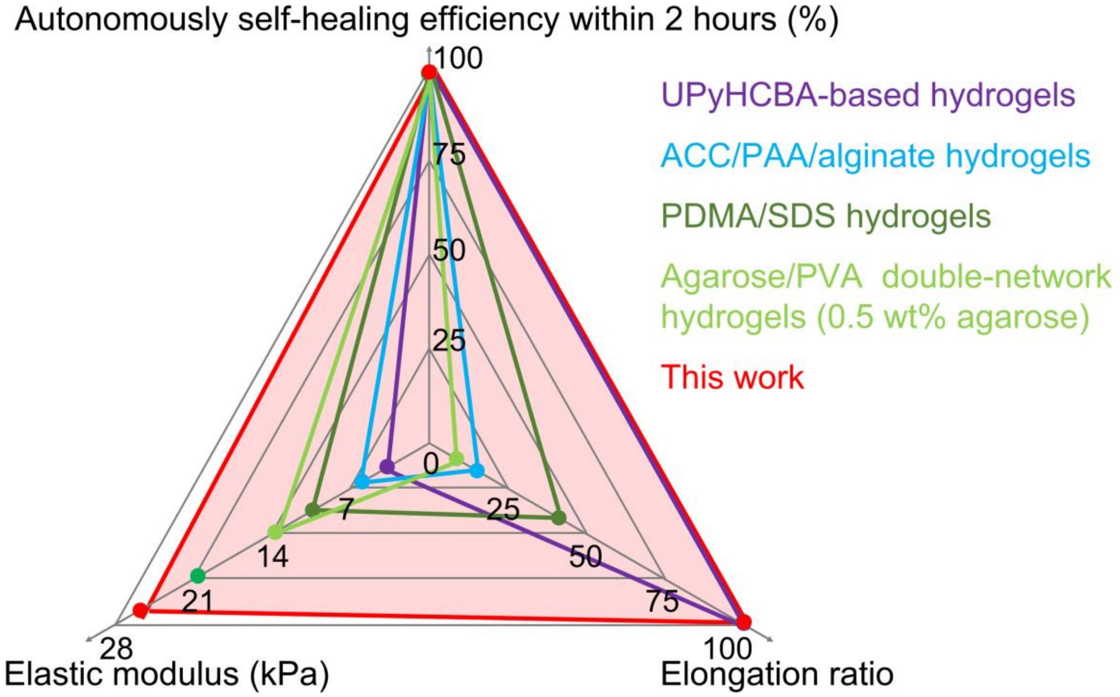

Supplementary Figure 13. A comparison of mechanical properties between this work and previously-reported stretchable and fast self-healing (within 2 hours) hydrogels.

Supplementary Figure 14

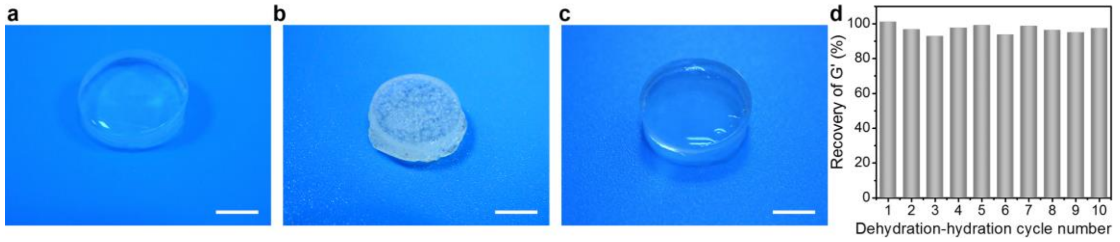

Supplementary Figure 14. The recyclability of the supramolecular polyelectrolyte hydrogel. (a) A photo of the original hydrogel. (b) A photo of the dehydrated porous xerogel. (c) A photo of the recovered hydrogel. (d) The quantitative recyclability assessment of the supramolecular polyelectrolyte hydrogel by the recovery of G' after multiple dehydration-hydration cycles. (Scale bar: 0.5 cm)

Supplementary Figure 15

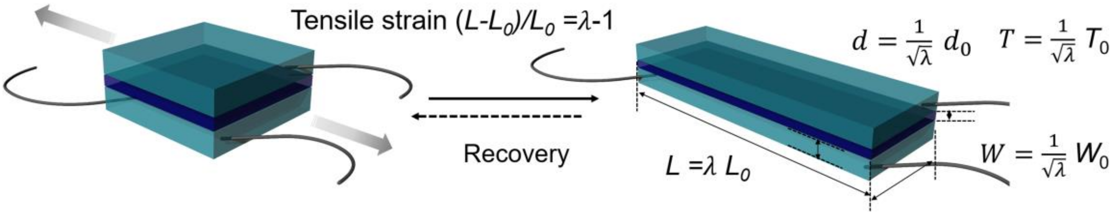

Supplementary Figure 15. Schematic illustration for the tensile strain sensor and the geometry changes during stretching.

Supplementary Figure 16

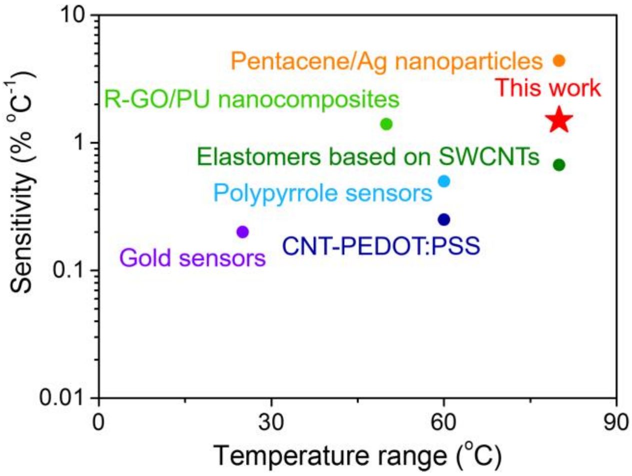

Supplementary Figure 16. A comparison of thermal sensory capability between this work and previously-reported skin-like thermal sensors.

Supplementary Figure 17

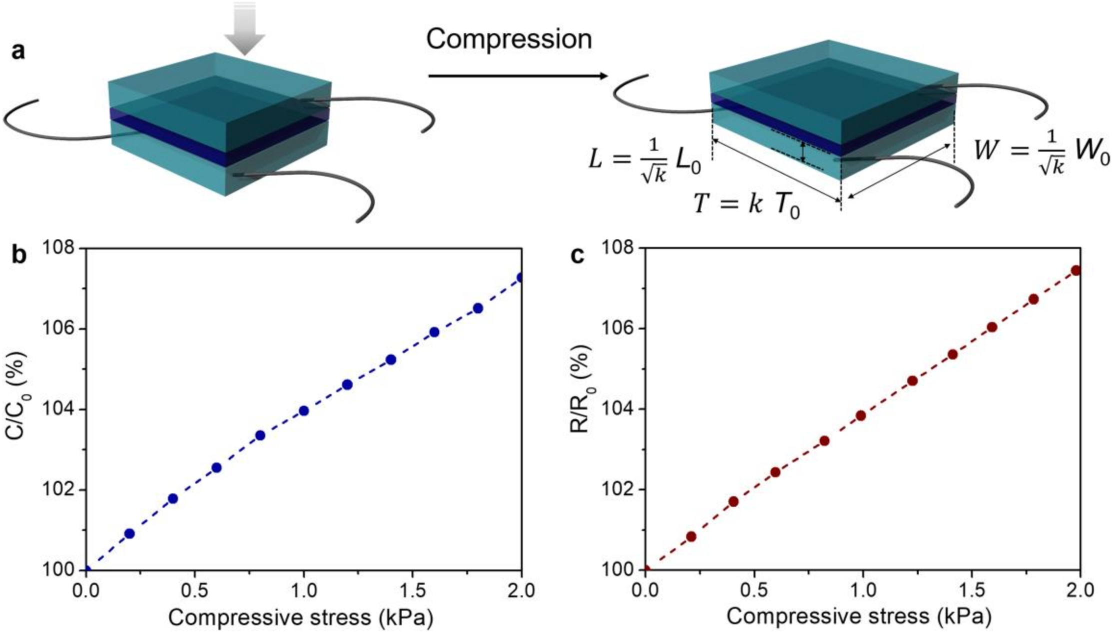

Supplementary Figure 17. Schematic illustration for the pressure sensor and the geometry, capacitance and resistance changes during compressing. (a) Schematic illustration. (b) The capacitance-stress relationship. (c) The resistance-stress relationship.

Supplementary Figure 18

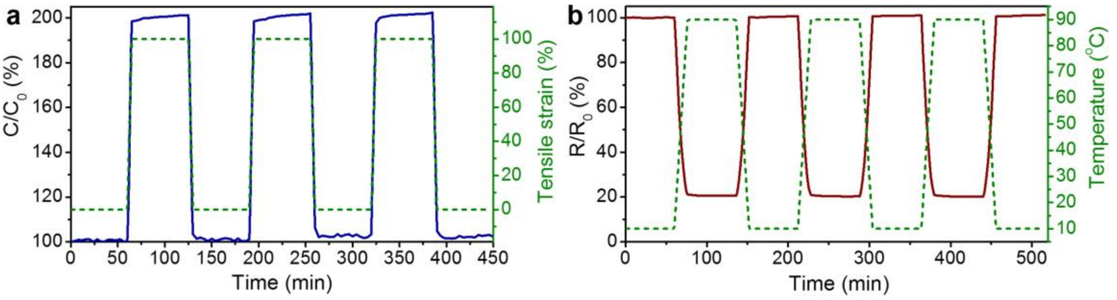

Supplementary Figure 18. Stepwise capacitance-strain and resistance-temperature changes. (a) Stepwise capacitance-strain changes in the range of 0-100% strain; (b) stepwise resistance-temperature changes in the range of 10-90 °C.

Supplementary Figure 19

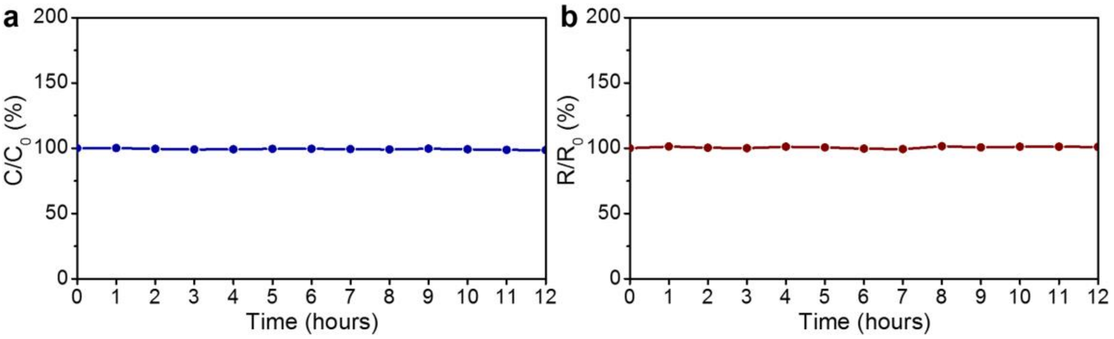

Supplementary Figure 19. Geometric stability of the hydrogel-based iontronics. (a) The capacitance change and (b) the resistance change of the supramolecular polyelectrolyte hydrogel which is placed in a closed environment (neither water evaporation nor epidermis-like VHB protection).

Supplementary Figure 20

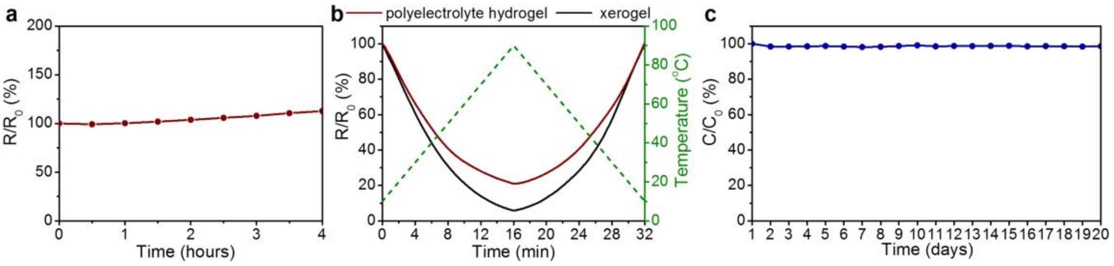

Supplementary Figure 20. Stability of the hydrogel-based iontronics exposed in a typical living environment. (a) The resistance change of the supramolecular polyelectrolyte hydrogel exposed in air with 60% relative humidity at 25 ^{o}C for 4 hours. (b) The resistance change and temperature curves of the polyelectrolyte hydrogel and dehydrated xerogel. (c) The capacitance change of the supramolecular polyelectrolyte hydrogel exposed in air with 60% relative humidity at 25 ^{o}C for 20 days.

Supplementary Figure 21

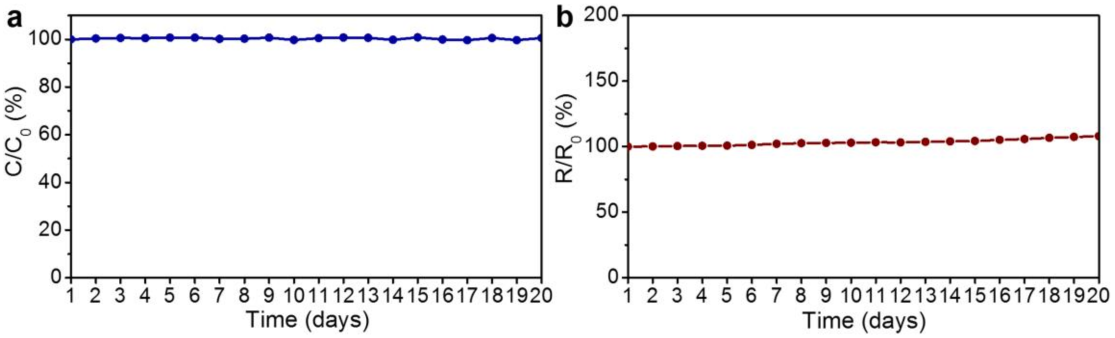

Supplementary Figure 21. Stability of the hydrogel-based iontronics protected by epidermis-like VHB tapes. (a) The capacitance change and (b) the resistance change of the hydrogel-based iontronics with epidermis-like VHB protection.

Supplementary Figure 22.

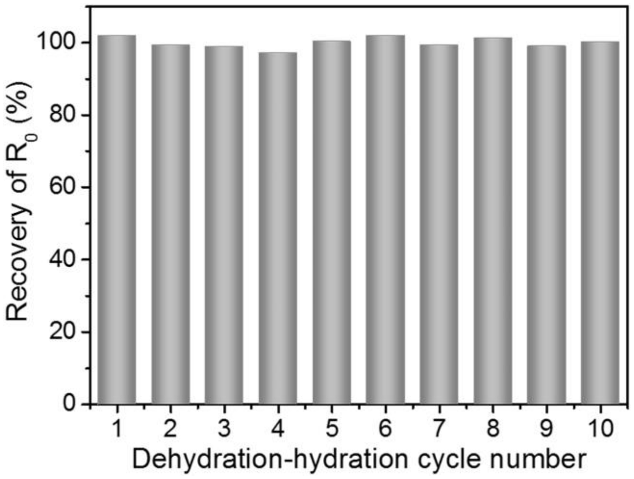

Supplementary Figure 22. The recovery of the resistance of the hydrogel during several dehydration-hydration cycles.

Supplementary Figure 23

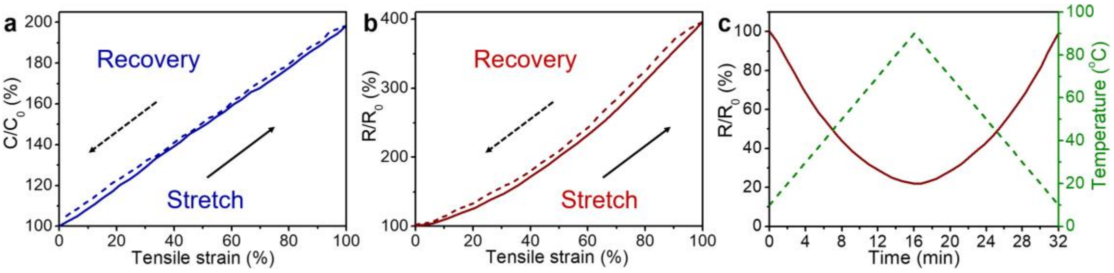

Supplementary Figure 23. When the damaged ionic-conductive hydrogel layer of the biomimetic skin heals itself, the sensory capabilities of the biomimetic skin also recover. (a) The capacitance-strain relationship after the self-healing of the ionic-conductive hydrogel layer. (b) The resistance-strain relationship after the self-healing of the ionic-conductive hydrogel layer. (c) The resistance-temperature relationship after the self-healing of the ionic-conductive hydrogel layer.

Supplementary Figure 24

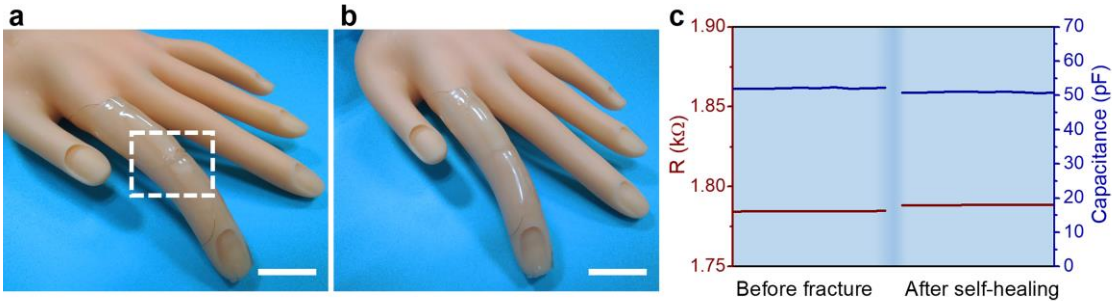

Supplementary Figure 24. When the commercial dielectric layer is also damaged, the restoration of sensory capabilities is further limited by the self-healability of the dielectric layer. (a) A photo of the fractured biomimetic skin. (b) A photo of the biomimetic skin after self-healing. (c) The resistance and capacitance of the biomimetic skin before fracture and after self-healing.

Supplementary Figure 25

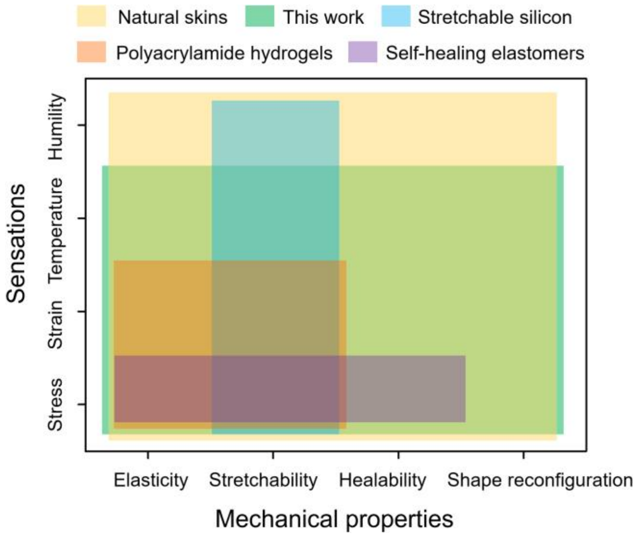

Supplementary Figure 25. A comparison of mechanical properties and sensations among natural skins, well-known artificial skins and this work.

Supplementary Movies 1-3

Supplementary Movie 1. A movie demonstrating the hydrogel's wide spectrum of mechanical properties (including robust elasticity, high stretchability, and shape reconfiguration).

Supplementary Movie 2. A movie showing a prosthetic finger which is compliantly covered with the transparent biomimetic skin can sense the finger's bending-release movements.

Supplementary Movie 3. A movie showing the prosthetic finger attached with the biomimetic skin can sense temperature stimulus by the contact with a person's hand and display real-time resistive response.

Supplementary References

1 Henderson, K. J. & Shull, K. R. Effects of solvent composition on the assembly and relaxation of triblock copolymer-based polyelectrolyte gels. *Macromolecules* **45**, 1631-1635 (2012).

2 Sun, T. L. *et al.* Bulk energy dissipation mechanism for the fracture of tough and self-healing hydrogels. *Macromolecules* **50**, 2923-2931 (2017).

3 Jeon, I., Cui, J., Illeperuma, W. R. K., Aizenberg, J. & Vlassak, J. J. Extremely stretchable and fast self-healing hydrogels. *Adv. Mater.* **28**, 4678-4683 (2016).

4 Wang, P., Deng, G., Zhou, L., Li, Z. & Chen, Y. Ultrastretchable, self-healable hydrogels based on dynamic covalent bonding and triblock copolymer micellization. *ACS Macro Lett.* **6**, 881-886 (2017).

5 Lei, Z., Wang, Q., Sun, S., Zhu, W. & Wu, P. A bioinspired mineral hydrogel as a self-healable, mechanically adaptable ionic skin for highly sensitive pressure sensing. *Adv. Mater.* **29**, 201700321 (2017).

6 Algi, M. P. & Okay, O. Highly stretchable self-healing poly(N,N-dimethylacrylamide) hydrogels. *Eur. Polym. J.* **59**, 113-121 (2014).

7 Chen, W.-P., Hao, D.-Z., Hao, W.-J., Guo, X.-L. & Jiang, L. Hydrogel with ultrafast self-healing property both in air and underwater. *ACS Appl. Mater. Interfaces*, **10**, 1258–1265 (2018).

8 Sun, J.-Y., Keplinger, C., Whitesides, G. M. & Suo, Z. Ionic skin. *Adv. Mater.* **26**, 7608-7614 (2014).

9 Webb, R. C. *et al.* Ultrathin conformal devices for precise and continuous thermal characterization of human skin. *Nature Mater.* **12**, 938-944 (2013).

10 Harada, S. *et al.* Fully printed flexible fingerprint-like three-axis tactile and slip force and temperature sensors for artificial skin. *ACS Nano* **8**, 12851-12857 (2014).

11 Yang, H. *et al.* Soft thermal sensor with mechanical adaptability. *Adv.*

Mater. **28**, 9175-9181 (2016).

12 He, Y., Gui, Q., Liao, S., Jia, H. & Wang, Y. Coiled fiber-shaped stretchable thermal sensors for wearable electronics. *Adv. Mater. Technol.* **1**, 1600170 (2016).

13 Trung, T. Q., Ramasundaram, S., Hwang, B.-U. & Lee, N.-E. An all-elastomeric transparent and stretchable temperature sensor for body-attachable wearable electronics. *Adv. Mater.* **28**, 502-509 (2016).

14 Ren, X. *et al.* A low-operating-power and flexible active-matrix organic-transistor temperature-sensor array. *Adv. Mater.* **28**, 4832-4838 (2016).

15 Jeon, J., Lee, H.-B.-R. & Bao, Z. Flexible wireless temperature sensors based on Ni microparticle-filled binary polymer composites. *Adv. Mater.* **25**, 850-855 (2013).

16 Yuk, H., Zhang, T., Parada, G. A., Liu, X. & Zhao, X. Skin-inspired hydrogel–elastomer hybrids with robust interfaces and functional microstructures. *Nature Commun.* **7**, 12028 (2016).

17 Kim, J. *et al.* Stretchable silicon nanoribbon electronics for skin prosthesis. *Nature Commun.* **5**, 5747 (2014).

18 Tee, B. C. K., Wang, C., Allen, R. & Bao, Z. An electrically and mechanically self-healing composite with pressure- and flexion-sensitive properties for electronic skin applications. *Nature Nanotechnol.* **7**, 825-832 (2012).
