# Supplementary material for: A supramolecular biomimetic skin combining a wide spectrum of mechanical properties and multiple sensory capabilities
Source: Nat Commun. 2018 Mar 19;9:1134. doi: 10.1038/s41467-018-03456-w (PMC5859265; doi:10.1038/s41467-018-03456-w)
Supplement: Supplementary file 2 — Description of Additional Supplementary Files(PDF 52 kb) [file 41467_2018_3456_MOESM2_ESM.pdf]

Description of Additional Supplementary Files

File Name: Supplementary Movie 1
Description: A movie demonstrating the hydrogel's wide spectrum of mechanical properties (including robust elasticity, high stretchability, and shape reconfiguration).

File Name: Supplementary Movie 2
Description: A movie showing a prosthetic finger which is compliantly covered with the transparent biomimetic skin can sense the finger's bending-release movements.

File Name: Supplementary Movie 3
Description: A movie showing the prosthetic finger attached with the biomimetic skin can sense temperature stimulus by the contact with a person's hand and display real-time resistive response.
